# Messrs. Bullock & Crenshaw’s Rejoinder to W. R. Warner & Co.

**Published:** 1878-01-20

**Authors:** 

**Affiliations:** Philadelphia


### MESSRS. BULLOCK & CRENSHAW'S REJOINDER TO W. R. WARNER & CO.

When we gave place, in our November number, to the article of Wm. R. Warner & Co., relative to sugar-coated pills, we did so to correct what we supposed to be a mistake in a mere matter of history, committed by our contributor, Dr. Greene. But Messrs. Bullock & Crenshaw send us the following rejoinder to the article referred to, and insist upon its publication, claiming a right to be heard. We publish it, but distinctly disclaim being a party to the controversy in any manner. The parties are our advertising patrons, and are both well and favorably known as among the first business men in the country.

We trust that no serious unpleasantness will result, and that the issues involved may find a satisfactory solution:

A CARD.

*Editors Southern Medical Record:*

Sirs—Our attention has been called to a quaint correspondence, published in the editorial columns of your November number, in which we regret to find our firm name used in such a manner as to demand an explanation from us. The article on Sugar-coated Pills, contributed by Dr. Wm. A. Greene, unsolicited by us, as you probably are aware, but was *entirely correct* in asserting that our firm commenced manufacturing S. C. Pills before any other individual or firm was similarly engaged in the State of Pennsylvania, and as this assertion conflicts materially with the statement made by Wm. R. Warner & Co., we desire to give, briefly, *the facts*, leaving it to your readers to determine whether our claim to priority in the *manufacture* of sugar-coated pills is correct or not.

About the year 1858, Mr. Warner was engaged in a strictly retail drug and prescription business in this city, with leisure time on his hands, while we were carrying on an extensive wholesale trade, extending far over this country, a large portion of which was with the physicians in the South. Not having sufficient room to spare for any new operations in our already crowded laboratory, we entered into an agreement with Mr. Warner to send to his store, from time to time, all the necessary ingredients, bottles, labels, wrappers, etc., he to employ suitable assistants, and return the Pills to us finished, charging us a stipulated price per thousand for the labor put upon them, which could have been done by any other pharmacist in the city, whom we might have selected or chosen to employ—all of the bottles, labels, wrappers, etc., used bearing the name of Bullock & Crenshaw as manufacturers, and Mr. Warner was not known in the matter. We commenced the advertisement of the pills in most of the medical journals of the country, and, by distributing the price-list, and assuming all the responsibility of manufacture and sale, after considerable expenditure, we succeeded in conquering the prejudices of the profession, and our sugar-coated pills became an indispensable luxury in every physician's outfit.

Mr. Warner asserts that he *supplied* B. & C. with sugar-coated pills for a period of eight years prior to 1866. We declare that he never *sold* us a pill during that period, but he was simply employed by us to work up our materials. As well might his assistant, whom he kept employed on our pills, claim to be the manufacturer. The whole responsibility was ours, the risk of reputation and capital was ours, and if the pills had proven a failure, we would have sustained the loss; but after the experiment had turned out a grand success, under our direction, in 1866, Mr. Warner entered into the wholesale business and commenced manufacturing sugar-coated pills. We secured a larger building, employed another pharmacist and assistants, and continued the manufacture of our pills without any delay or interruption.

In regard to the awards of the Centennial Commission, we notice that Messrs. Warner & Co. inform your reader that they received their diploma and medal from the "primary" judges, and that B. & C. received theirs from a "supplementary" jury, appointed by the commission near the close of the exhibition—all of which is entirely correct—but we regret that they did not complete the narrative, and anticipate the questions which will naturally arise in the minds of every thinking man, as to *why* the commission deemed it necessary to *supplement* the action of the "primary" board.

The Centennial Commission was comprised of honest, intelligent, and prudent men from all parts of the country, and they certainly would not have deemed it necessary to appoint a "supplementary" jury, just before the close of the exhibition, without sufficient cause to justify such action. The conclusions arrived at by the "primary" board must have been very unsatisfactory to compel the commission to resort to the unusual proceeding of appointing a "supplementary" jury. We are glad to be able to state that our diploma and medal were awarded by the "supplementary" jury, signed by Professor Henry H. Smith, M.D., of Philadelphia, and not by the "primary" board, in which Dr. J. H. Thompson, of Washington, was so active.

Very respectfully,
BULLOCK & CRENSHAW.

*Philadelphia, Dec.* 22, 1877. (1t)

